# Chemical Composition, Repellency, and Insecticidal Activity of *Pinus halenpenssis* Leaf Essential Oil from Morocco on Adults of *Rhyzopertha dominica* (Fabricius) (Coleoptera: Bostrichidae) and *Tribolium castaneum* (Herbst) (Coleoptera: Tenebrionidae)

**DOI:** 10.3390/plants14030407

**Published:** 2025-01-30

**Authors:** Imane Naimi, Hafida Bouamama, Touria Ba M’hamed

**Affiliations:** Laboratory of Sustainable Development and Health Research, Faculty of Sciences and Techniques, Cadi Ayyad University, Marrakech 40000, Morocco

**Keywords:** *R. dominica*, *T. castaneum*, *P. halepensis*, EO, repellency, toxicity

## Abstract

*Rhyzopertha dominica* and *Tribolium castaneum* are two significant insect pests that affect the quality, quantity, and commercial value of stored products. The aim of this study was to assess the chemical composition, repellency, and insecticidal activity of *Pinus halepensis* leaf EO on adults of *Rhyzopertha dominica* (Fabricius) (Coleoptera: Bostrichidae) and *Tribolium castaneum* (Herbst) (Coleoptera: Tenebrionidae). The EO of *P. halepensis* Mill. was extracted using hydrodistillation and analyzed for its chemical composition by GC-MS. The major components identified were 1-nonadecene (25.51%), 1-hexadecene (20.79%), pimaric acid (16.71%), and palmitic acid (12.47%). The repellency test was determined by the area-preference method. *P. halepensis* EO exhibited significant repellent activity against *T. castaneum* and *R. dominica*. It showed high class IV repulsion rates, reaching 63.60% against *T. castaneum* and 66.50% against *R. dominica*. The repellent effect was most potent at the highest concentration tested (16 µL/mL), achieving a 100% efficacy against *T. castaneum* after 4 h and after 3 h against *R. dominica*. The contact toxicity test was carried out by impregnating filter paper disks with increasing doses of the EO studied. *P. halepensis* EO was the most toxic against *R. dominica* (LC_50_ = 17.11 µL/mL, LC_90_ = 30.02 µL/mL) and *T. castaneum* (LC_50_ = 20.92 µL/mL, LC_90_ = 32.18 µL/mL) after 96 h of exposure. The ability of *P. halepensis* EO to repel and eliminate insects suggests that it could be used as a new treatment to prevent insect infestations of *R. dominica* and *T. castaneum*.

## 1. Introduction

Insect pests pose a significant threat to food that is stored. Their attacks result in substantial financial losses for consumers, food companies, and farmers. The presence of these insects in food products can compromise their sanitary quality, thereby posing a risk to public health [1]. The largest group of insects is beetles, which have over 250,000 species. The most dangerous species for warehouses are those in one of the following families: Tenebrionidae, Bostrichidae, Bruchidae, Cucujidae, Curculionidae, Dermestidae, and Silvanidae [2,3]. Two major pests of stored grains are *Rhyzopertha dominica* (Fabricius) (Coleoptera: Bostrichidae) and *Tribolium castaneum* (Herbst) (Coleoptera: Tenebrionidae) [4]. The implementation of effective measures for the prevention and control of insect pests in food products is of paramount importance to guarantee the availability of safe and healthy food for the world population [5,6]. Chemical control is the most widely used method to protect stored products. The advantages of this practice are linked to the ease of implementation and the duration of protection, which can last several months. Unfortunately, the application of chemical insecticides is limited by many constraints, which are their high cost, the accumulation of toxic residues, and the development of insecticide resistance [7,8,9]. Consequently, it is essential to create safe and environmentally friendly alternatives to chemical insecticides [10]. Many plants exhibit a variety of biological activities against insects and other organisms, yet their chemical composition determines their efficacy [11,12]. Essential oils (EOs) are volatile compounds extracted from aromatic and medicinal plants. They act on different aspects of the life of insects, such as survival, oviposition (laying eggs), reproduction, longevity, and other developmental parameters [13,14]. EOs can be evaluated for repellent activity as well as the toxicity test through contact, fumigation, and ingestion, providing natural, biodegradable, and less harmful alternatives to synthetic chemical insecticides for the environment and human health [15,16,17]. Plant extracts primarily target harmful insects via neurotoxic effects such as acetylcholinesterase, which causes insect death [18]. *Pinus halepensis* Mill. is a coniferous tree in the Pinaceae family, also known as Aleppo pine. Additionally, it is a Mediterranean medicinal plant that has a wide range of traditional applications, such as treating diarrhea, wounds, rheumatism, cough, gastrointestinal disorders, hypertension, and hemorrhoids [19,20,21,22]. In the context of the biological control of agricultural pests, the study of the chemical composition, repellency, and insecticidal activity of *P. halepensis* leaf EO on adults of *Rhyzopertha dominica* (Fabricius) (Coleoptera: Bostrichidae) and *Tribolium castaneum* (Herbst) (Coleoptera: Tenebrionidae) constitutes an original and innovative contribution. This study’s significance lies in the exploration of the biochemical properties of this plant to provide sustainable alternatives to synthetic pesticides, an approach which is all the more relevant in a context where agriculture is looking for more environmentally friendly solutions.

## 2. Results

### 2.1. Yield and Chemical Composition of the EO

The yield of *P. halepensis* EO is recorded as 0.43% (*w*/*w*), and the chemical composition of the EO is presented in Table 1. A total of 16 compounds were identified, with 1-nonadecene (25.51%), 1-hexadecene (20.79%), pimaric acid (16.71%), and palmitic acid (12.47%) as the major compounds. Minor compounds included lauric acid (0.93%), eucalyptol (0.90%), and palmitoleic acid (0.88%). The EO was characterized by the presence of unsaturated hydrocarbon “alkenes” (53.06%), diterpenes (18.05%), saturated fatty acids (15.61%), monoterpenes (3.14%), unsaturated fatty acids (3.61%), sesquiterpenes (2.24%), saturated hydrocarbon “alkanes” (2.01%), and alcohol (1.72%).

### 2.2. Repellency of EO

**Figure 1:**
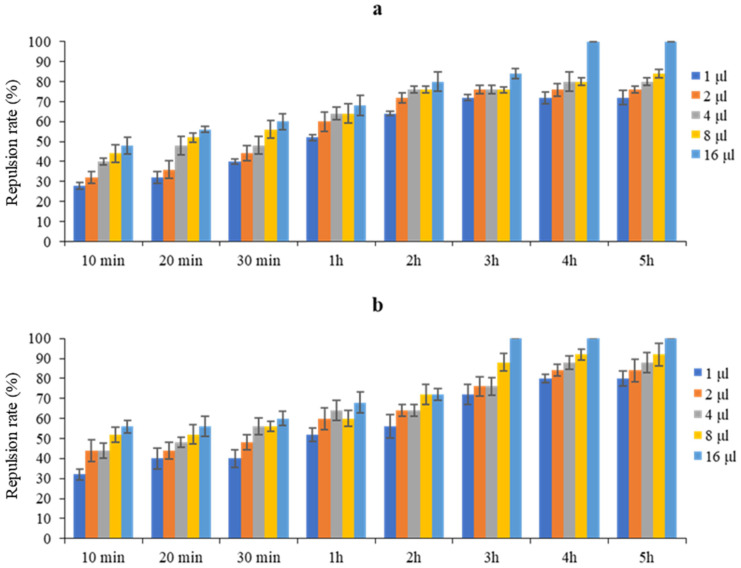
Repellent activity of *P. halepensis* EO against *T. castaneum* (**a**) and *R. dominica* (**b**) at different doses and exposure times.

Figure 1 shows that the dose of 16 µL/mL exhibited the highest repellent effect, reaching 100% against *T. castaneum* after 4 h and remaining stable at this level for up to 5 h. Similarly, for *R. dominica*, the dose of 16 µL/mL also showed a high repellent effect, reaching 100% after only 3 h and stabilizing for up to 5 h. These results show that the EO tested at a dose of 16 µL/mL proved to be particularly effective in repelling both *T. castaneum* and *R. dominica*.

Table 2 shows that the different doses of *P. halepensis* EO from 1 to 16 µL/mL induced repulsion rates ranging from 54.00 to 74.50% against *T. castaneum*, with an average rate of 63.60%, and from 56.50 to 76.50% against *R. dominica*, with an average rate of 66.50%. This suggests that *P. halepensis* EO exhibited class IV repellency towards both insects.

### 2.3. Contact Toxicity of EO

The contact toxicity of *P. halepensis* EO against *T. castaneum* and *R. dominica* is given in Table 3. For *T. castaneum*, the mortality rate increased progressively, ranging from 42% at 24 h to 63% at 96 h. In contrast, *R. dominica* showed a significantly higher sensitivity, with an initial mortality rate of 54% at 24 h which increases significantly to reach 72% after 96 h. *P. halepensis* EO showed the highest contact toxicity with LC_50_ values of 20.92 µL/mL and LC_90_ values of 32.18 µL/mL against *T. castaneum* and LC_50_ values of 17.11 µL/mL and LC_90_ values of 30.02 µL/mL against *R. dominica* after 96 h of exposure.

## 3. Materials and Methods

### 3.1. Rearing of Insects

*R. dominica* and *T. castaneum* were collected from several infested products (wheat grain, wheat flour, and semolina). The mass rearing of each insect was conducted in glass jars with 500 g of semolina, previously frozen for 24 h before being introduced into the jars to destroy the eggs of other insects possibly present. This made it possible to obtain a large number of insects while ensuring the homogeneity of the populations tested. These jars were kept in incubators set at a temperature of 25 ± 2 °C, a relative humidity of 60 ± 5%, and a photoperiod 10:14 h (light/dark) to optimize the development of the insects. The experiments employed unsexed insects of *R. dominica* (aged 3 to 5 days) and *T. castaneum* (aged 7 to 10 days) under identical rearing conditions.

### 3.2. Plant Material and EO Extraction

The leaves of *P. halepensis* were collected in April 2022 from the botanical garden at the Faculty of Sciences and Techniques, Cadi Ayyad University, Marrakech, air-dried, and subjected to hydrodistillation for 4 h using a Clevenger-type apparatus. The obtained EO was dried with anhydrous sodium sulfate (Na_2_SO_4_). The yield of EO was calculated (EO_Y_) using the equation of Fakayode and Abobi [23]:

(1) $${EO}_{Y}\left(\%\right)=\frac{M_{f}-M_{e}}{M_{s}}\times 100$$

where **EO_Y_** is the EO yield (%), **M_f_** is the mass of the flask and extracted essential oil (g), **M_e_** is the mass of the empty flask (g), and **M_s_** is the mass of sample (g).

### 3.3. Chemical Analysis by Gas Chromatography–Mass Spectrophotometry (GC-MS)

The chemical composition of the EO was analyzed by gas chromatography coupled to mass spectrometry (GC-MS) using HP 5973 equipped with a capillary column DB-5 (30 m × 0.25 mm and 0.25 μm film thickness). The mass spectra were recorded at 70 eV. The column temperature program was 60 °C for 5 min, then ramped up to 3 °C/min from 60 °C to 240 °C, and finally 10 °C/min from 240 °C to 300 °C, where it was held for 5 min. A volume of 1 µL of each sample was injected using a split mode (1/70). The helium was the carrier gas used at a flow rate of 2 mL/min. Identifying the components of the EO was achieved by comparing the mass spectra of the EO components with the National Institute of Standards and Technology (NIST) Mass Spectral Library.

### 3.4. Repellency Assay of EO

**Figure 2:**
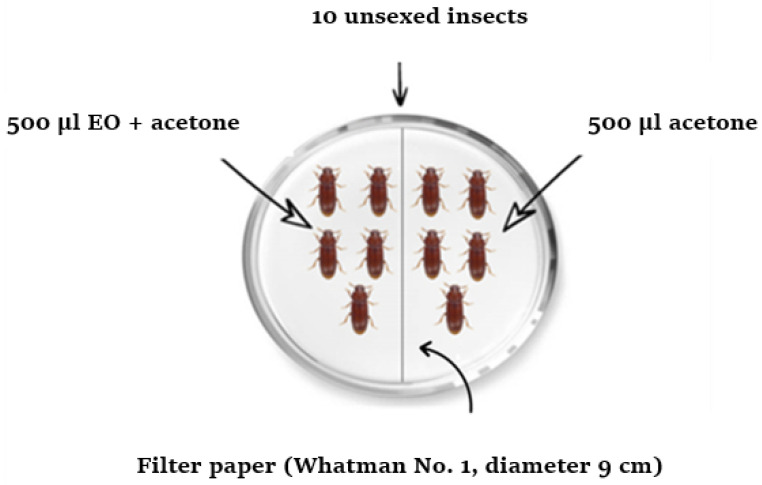
Experimental design for assessing the repellent activity of the EO.

The assay of the repulsive activity of the EO against *T. castaneum* and *R. dominica* was carried out in glass Petri dishes (9 cm in diameter) using the zone-choice tests described by McDonald et al. [24]. Solutions were prepared by diluting each one of the following five amounts of EO (1, 2, 4, 8, and 16 µL) in 1 mL of acetone. In Petri dishes, Whatman N° filter papers (9 cm in diameter) were cut in half: one half was treated with 0.5 mL of the appropriate concentration of EO and the other with 0.5 mL of acetone (Figure 2). The treated and control halves were air-dried for 15 min to evaporate the solvent and subsequently placed side-by-side in a Petri dish. A group of ten non-sexed adults of either insect was introduced in the center of each Petri dish. Each concentration was replicated five times. The treated Petri dishes were held under conditions identical to those used for mass breeding, described previously. The total number of insects present on the treated and control halves of the filter papers was counted and recorded after 10 min, 20 min, 30 min, 1 h, 2 h, 3 h, 4 h, and 5 h of exposure. The percentage of repulsion (PR) was calculated for each concentration and at each exposure time using the formula of McDonald et al. [24].

(2) $$PR=\frac{N_{t}-N_{c}}{N_{t}+N_{c}}\times 100$$

where **N_t_** is the total number of insects present in the treated half, and **N_c_** is the total number of insects present in the control half.

The PR values were assigned to one of the following repulsive classes, ranging from 0 to V, using the scale described by McDonald et al. (1970): class 0 (PR < 0.1%), non-repellent; class I (PR: 0.1–20.0%), very weakly repellent; class II (PR: 20.1–40.0%), weak repellent; class III (PR: 40.1–60.0%), moderately repellent; class IV (PR: 60.1–80.0%), repellent; and class V (PR: 80.1–100.0%), very repellent.

The repellency index (RI) was calculated using the formula of Mazzonetto [25]:

(3) $$RI=\frac{2G}{G+P}$$

where **G** is the percentage of insects present in the treated area, and **P** is the percentage of insects present in the untreated area.

The RI indicates whether a chemical substance is repelling, attracting, or neutral to a tested insect [26]:

- RI < 1—the substance is classified as a repellent, indicating that insects avoid the treated area;
- RI = 1—the substance is neutral, with no observable effect on insect distribution;
- RI > 1—the substance is attractive, indicating an insect preference for the treated area.

### 3.5. Contact Toxicity Assay of EO

**Figure 3:**
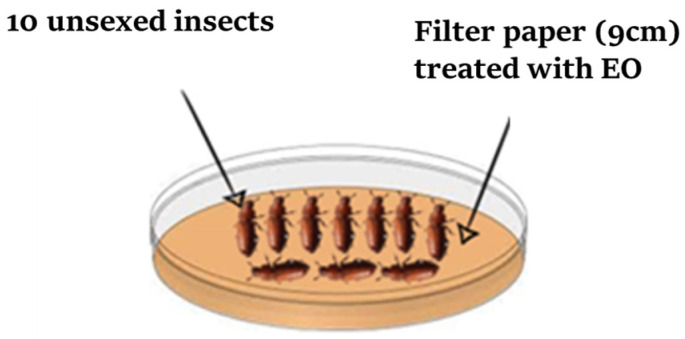
Experimental design for assessing the contact toxicity of the EO.

The assay to determine the contact toxicity of the EO against *T. castaneum* and *R. dominica* was carried out in glass Petri dishes (9 cm in diameter) according to the method described by Ebadollahi et al. [27]. Each one of the following five amounts of EO (15, 20, 25, 30, and 35 µL) was diluted in 1 mL of acetone and distributed uniformly on individual filter paper disks (Whatman No. 1 of 9 cm in diameter). A control filter paper disk was treated only with 1 mL of acetone (Figure 3). All disks were left at ambient temperature for 15 min to allow for the complete evaporation of the dilution solvents. A treated or control filter paper was placed individually in a Petri dish, and ten adults of each insect were released in the center of each Petri dish, which was immediately covered. Each EO concentration was tested five times. The number of dead insects was recorded daily after each 24 h period, and the percentage of mortality was calculated and corrected using the Abbott formula [28].

(4) $$M_{c}=\frac{M_{O}-M_{t}}{100-M_{t}}\times 100$$

where **M_o_** is the mortality observed in the treated Petri dishes, **M_t_** is the mortality observed in the control, and **M_c_** is the calculated mortality.

### 3.6. Statistical Analysis

The results of the tests performed are hereby expressed as the mean ± standard error (SE). Differences between the measured variables were analyzed using ANOVA with a post hoc Tukey HSD test. The percentage of mortality was subjected to Probit analysis to calculate the LC_50_ and LC_90_ at 95% of the fiducial limits of the upper and lower confidence limits. Statistical analysis was performed using SPSS software version 25.0, with *p* < 0.05 indicating statistical significance.

## 4. Discussion

EOs, which are also referred to as volatile oils or essences, are secondary metabolites that are produced by aromatic plants. These compounds are essential for their defense system against microorganisms, insects, and other harmful agents [29,30].

*P. halepensis* EO exhibits significant repellent activity against *T. castaneum* and *R. dominica*. It showed high class IV repulsion rates, reaching 63.60% against *T. castaneum* and 66.50% against *R. dominica*. The RI values, which were less than 1 for all concentrations applied over time, ranging from 10 min to 5 h, confirm these results and the ability of EO to repel both insects. The fact that the insects avoided the treated half-disks and moved more quickly toward the second half (control) demonstrated the repellent activity of this EO. These results are in agreement with previous studies showing the repellent effect of various EOs, which impact the movement behavior of insect pests present in stored products [31,32,33]. The potential degree of insect response is related to the chemical composition and concentration of the applied EO [34]. According to Torto [35], insects exposed to chemical signals exhibit selective responses to the active components of the signal. The chemical composition of the tested EO revealed the presence of unsaturated hydrocarbon “alkenes” (53.06%), diterpenes (18.05%), saturated fatty acids (15.61%), monoterpenes (3.14%), unsaturated fatty acids (3.61%), sesquiterpenes (2.24%), saturated hydrocarbon “alkanes” (2.01%), and alcohol (1.72%). Previous studies have shown that the repellent properties of EOs are characterized by the presence of monoterpenoids, sesquiterpenes, saturated and unsaturated fatty acids, and alcohols [36,37,38,39,40], According to Green [36], unsaturated fatty acids, like palmitoleic and linolenic acids, are more volatile and can repel *Liposcelis bostrychophila*. Saturated fatty acids, including palmitic acid, lauric acid, and myristic acid, exhibit effective repellent properties against *Spodoptera littoralis* (Boisd.) and *Plutella xylostella* (L.) [40,41]. The repellent effect of *P. halepensis* EO against *T. castaneum* and *R. dominica* can also be attributed to the presence of monoterpenes (eucalyptol and camphor) and sesquiterpenes (α-epi-Cadinol). A multitude of monoterpenes and sesquiterpenes have been investigated for their insect-repellent properties [42,43]. Additionally, Zhang et al. [44] indicated that the potent repellent effect of EOs may result from the synergistic interactions of their various major and minor compounds. Furthermore, our results demonstrated that the repellency increased with increasing concentrations and exposure times. The repellent effect was most potent at the highest concentration tested (16 µL/mL), achieving a 100% efficacy against *T. castaneum* after 4 h and remaining stable at this level for up to 5 h. The same concentration (16 µL/mL) demonstrated a high repellent effect for *R. dominica*, which reached 100% after only 3 h and remained stable for up to 5 h. These findings suggest that this concentration is adequate to guarantee the complete repulsion of both species, with *R. dominica* exhibiting a slightly higher sensitivity. The repulsive effectiveness of an EO also depends on the specific sensitivity of each insect exposed, more precisely, the sensitivity of the olfactory receptors of the neurons located on the antennae to certain components of the EO more than others and to the specific characteristics of the compounds and their individual concentrations, which act in different ways on different insects [45,46,47,48]. The repulsive action and persistence of EOs are influenced by the size, shape, and assembly of each compound’s active molecules and their persistence in insect antennae’s sensory receptors [46,49]. The increased sensitivity of *R. dominica* could be attributed to differences in the architecture of the odorant-binding site, specifically the structure and function of its chemical receptors, which allow it to detect and react more intensively to repulsive volatile molecules [50,51]. In addition, an insect’s response to plant odors is also affected by its age, motivation, and physiological state [52]. According to Concho et al. [53], insects must respond to instantaneous odor concentration changes, as well as the speed and precision of their olfactory system, when detecting an odor source. A wide variety of odorant receptors on the ciliated dendritic terminals of olfactory sensory neurons facilitate this form of detection [54]. Repulsion alters the perception of the peripheral nervous system (PNS), which causes the insect to move away from its food source [55]. Moreover, at high concentrations, volatile compounds appear to saturate odorant receptors, which may explain the stability of the repulsion rate.

The EO of *P. halepensis* was found to be the most toxic by contact, with the lowest LC_50_ and LC_90_ values for the two insects studied. *T. castaneum* had an LC_50_ of 20.92 µL/mL and an LC_90_ of 32.18 µL/mL. *R. dominica* had a lower LC_50_ of 17.11 µL/mL and an LC_90_ of 30.02 µL/mL after 96 h of exposure. The effectiveness of the EO could be attributed to its chemical composition [56]. This activity could result from the presence of oxygenated monoterpenes such as eucalyptol (0.9%) and camphor (2.24%), recognized for their powerful insecticidal action [57]. The nature and concentrations of the compounds could also contribute to this difference in activity [58,59]. Ejjabraoui et al. [60] confirmed these results by demonstrating the insecticidal activity of two Moroccan pine species, *Pinus halepensis* and *Pinus pinaster*, against *Bruchus signaticornis* (Coleoptera: Chrysomelidae). According to Quintai and Yongcheng [61], the contact activity of camphor demonstrated an insecticidal effect against the adults of *T. castaneum* and *R. dominica,* causing 78.5% mortality, but only at the concentration of 10.0 µL and the prolonged exposure time of 24 h. Furthermore, Wu et al. [62] reported that camphor and eucalyptol showed strong contact toxicity against *Lasioderma serricorne* (Coleoptera: Anobiidae). In the case of contact toxicity, these monoterpene compounds can penetrate through the cuticle to act on insects [63]. This contact effect promotes their absorption and increases their toxicity. According to Chaudhari et al. [64], these compounds can quickly penetrate the insect’s nervous system, thus accentuating their neurotoxic effect, which leads to paralysis or death in the insects affected. Certain compounds present in EOs may inhibit acetylcholinesterase, an enzyme essential for nerve transmission, as demonstrated by Colovic et al. [65] and Chaubey [66]. Eucalyptol, for instance, is neurotoxic because it blocks the octopamine receptor, which disturbs nerve signals and kills insects [67,68]. Moreover, fatty acids such as palmitoleic acid, linolenic acid, palmitic acid, lauric acid, and myristic acid have shown insecticidal activity against insects [40,69,70,71]. The results of the current study also showed that the adults of *R. dominica* are more sensitive to EO compared to those of *T. castaneum*. Our results are in agreement with those of Toews and Subramanyam [72], who showed that *R. dominica* was the most sensitive to spinosad, followed by *T. castaneum*. Toews and Subramanyam [72] suggest that *R. dominica’s* sensitivity could be due to its faster penetration through the insect’s cuticle and/or the tarsal pathway, increased sensitivity at the target site, or a reduction in metabolic detoxification. The cuticle, the protective barrier of insects, plays a crucial role in their sensitivity to insecticides. Its thickness and composition directly influence the penetration of insecticidal molecules and the activity of detoxification enzymes [73,74,75,76]. The constituents of essential oils, by diffusing through the cuticle, can weaken this barrier and facilitate the penetration of toxic substances, leading to the death of the insect [77,78].

## 5. Conclusions

The current research revealed that *P. halepensis* EO exhibits repellent and insecticidal effects against *R. dominica* and *T. castaneum*. Several factors can influence this effectiveness, like its specific chemical composition, the interactions among the compounds, the concentrations applied, the exposure period, and the targeted insect. High concentrations are more effective in repelling and killing the two studied species. *R. dominica* demonstrated a higher sensitivity than *T. castaneum*, especially over long periods of exposure. Over prolonged periods, repellency against *R. dominica* remains high even at moderate concentrations, while *T. castaneum* requires higher concentrations to maintain a sustained effect. The ability of *P. halepensis* EO to repel and eliminate insects suggests that it could be used as a new treatment to prevent insect infestations of *R. dominica* and *T. castaneum*.

## Figures and Tables

**Table 1:** Chemical composition of the EO of *P. halepensis*.

N°	RT ^a^ (min)	Compounds	Molecular Formula	Percentage (%)
1	5.59	Eucalyptol	C_10_H_18_O	0.9
2	6.66	Camphor	C_10_H_16_O	2.24
3	7.24	Hexadecane	C_40_H_82_O_2_	2.01
4	7.71	Linolenic acid	C_18_H_30_O_2_	2.73
5	8.00	Palmitoleic acid	C_16_H_30_O_2_	0.88
6	9.16	Geranyl-α-terpinene	C_20_H_32_	1.34
7	9.34	1-Heptatriacotanol	C_37_H_76_O	1.72
8	9.69	1-Hexadecene	C_16_H_32_	20.79
9	10.02	Lauric acid	C_12_H_24_O_2_	0.93
10	10.10	α-epi-Cadinol	C_15_H_26_O	2.24
11	10.80	1-Nonadecene	C_19_H_38_	25.51
12	11.10	Myristic acid	C_14_H_28_O_2_	2.21
13	12.05	Palmitic acid	C_16_H_32_O_2_	12.47
14	12.73	1-Docosene	C_22_H_44_	4.80
15	13.11	Pimaric acid	C_20_H_30_O_2_	16.71
16	14.38	17-Pentatriacontene	C_35_H_70_	1.96
Total identified (%)				99.44

^a^ RT: Retention time.

**Table 2:** Average repellency rate and index of *P. halepensis* EO against adults of *T. castaneum* and *R. dominica*.

Insects	Doses (μL/mL)	PR ± SE	Repellency Class	RI ± SE	Classification
*T. castaneum*	1	54.00 ± 4.00 ^e^	Class III	0.46 ± 0.08 ^e^	Repellent
	2	59.00 ± 3.82 ^d^	Class III	0.40 ± 0.03 ^d^	Repellent
	4	64.00 ± 5.00 ^c^	Class IV	0.37 ± 0.02 ^c^	Repellent
	8	66.50 ± 3.00 ^b^	Class IV	0.33 ± 0.05 ^b^	Repellent
	16	74.50 ± 3.91 ^a^	Class IV	0.26 ± 0.04 ^a^	Repellent
	Mean ± SD	63.60 ± 2.89 ^b^	Class IV	0.36 ± 0.01 ^c^	Repellent
*R. dominica*	1	56.50 ± 8.00 ^e^	Class III	0.44 ± 0.02 ^e^	Repellent
	2	63.00 ± 6.72 ^d^	Class IV	0.37 ± 0.05 ^d^	Repellent
	4	66.00 ± 5.00 ^c^	Class IV	0.35 ± 0.07 ^c^	Repellent
	8	70.50 ± 4.47 ^b^	Class IV	0.30 ± 0.05 ^b^	Repellent
	16	76.50 ± 3.49 ^a^	Class IV	0.23 ± 0.06 ^a^	Repellent
	Mean ± SD	66.50 ± 1.70 ^c^	Class IV	0.34 ± 0.02 ^bc^	Repellent

PR: percentage of repulsion; and RI: repellency index. PR and RI values were calculated for each concentration and at all exposure times, ranging from 10 min to 5 h. These values are expressed as a mean ± standard error (n = 5). Different letters in one column indicate a significant difference (*p* < 0.05).

**Table 3:** Contact toxicity of *P. halepensis* EO against adults of *T. castaneum* and *R. dominica*.

Insects	Time (h)	Mortality Rate ± SE	LC_50_ (μL/mL) [CI 95%]	LC_90_ (μL/mL) [CI 95%]
*T. castaneum*	24	42.00 ± 1.80	29.32 (18.63–33.33)	49.04 (42.93–68.05)
	48	48.00 ± 1.20	26.46 (12.85–30.66)	46.17 (40.07–55.14)
	72	57.00 ± 1.49	23.02 (9.86–31.55)	34.73 (31.61–37.08)
	96	63.00 ± 0.87	20.92 (7.38–29.89)	32.18 (27.28–34.79)
*R. dominica*	24	54.00 ± 2.90	23.47 (15.77–26.08)	38.35 (36.51–198.88)
	48	60.00 ± 1.68	21.62 (14.18–28.11)	34.22 (31.74–81.73)
	72	66.00 ± 2.00	19.97 (13.23–24.36)	32.50 (27.48–66.40)
	96	72.00 ± 1.76	17.11 (9.03–16.73)	30.02 (23.98–57.24)

CI: 95% confidence interval. The values of the mortality rate are expressed as a mean ± standard error (n = 5).

## Data Availability

The data presented in this study are available upon request from the corresponding author.

## References

[1] Abrol D.P., Shankar U. (2014). Pesticides, food safety and integrated pest management. Integrated Pest Management.

[2] Canhilal R. (2016). The use of entomopathogens in the controlling of insect pests of stored product. Sci. Pap. Ser. A Agron..

[3] Rees D.P. (2018). Coleoptera. Integrated Management of Insects in Stored Products.

[4] Yasir M. (2018). Repellent potential of three medicinal plant extracts against *Tribolium castaneum* (Coleoptera: Tenebrionidae). Punjab Univ. J. Zool..

[5] Sileem T.M., Mehany A.L., Hassan R.S. (2019). Fumigant toxicity of some essential oils against Red Flour Beetles, *Tribolium castaneum* (Herbst) and its safety to mammals. Braz. J. Biol..

[6] Mssillou I., Agour A., Allali A., Saghrouchni H., Bourhia M., El Moussaoui A., Salamatullah A.M., Alzahrani A., Aboul-Soud M.A., Giesy J.P. (2022). Antioxidant, Antimicrobial, and Insecticidal Properties of a Chemically Characterized Essential Oil from the Leaves of *Dittrichia viscosa* L.. Molecules.

[7] Fianko J.R., Donkor A., Lowor S.T., Yeboah P.O., Glover E.T., Adom T., Faanu A. (2011). Health risk associated with pesticide contamination of fish from the Densu River Basin in Ghana. J. Environ. Prot..

[8] Pretty J., Hine R. (2012). Pesticide use and the environment. The Pesticide Detox.

[9] Aboelhadid S.M., Youssef I.M. (2021). Control of red flour beetle (*Tribolium castaneum*) in feeds and commercial poultry diets via using a blend of clove and lemongrass extracts. ESPR.

[10] Rajashekar Y., Gunasekaran N., Shivanandappa T. (2010). Insecticidal activity of the root extract of *Decalepis hamiltonii* against stored-product insect pests and its application in grain protection. JFST.

[11] Yuan Z., Hu X.P. (2012). Repellent, antifeedant, and toxic activities of *Lantana camara* leaf extract against *Reticulitermes flavipes* (Isoptera: Rhinotermitidae). J. Econ. Entomol..

[12] Abbas M.G., Haris A., Binyameen M., Nazir A., Mozūratis R., Azeem M. (2022). Chemical composition, larvicidal and repellent activities of wild plant essential oils against *Aedes aegypti*. Biology.

[13] Reddy D.N. (2019). Essential oils extracted from medicinal plants and their applications. Natural Bio-Active Compounds.

[14] de Paiva Silva G.T., Figueiredo K.G., Alves D.S., de Oliveira D.F., Silva G.H., de Souza e Silva G.T., de Oliveira M.S., Biondi A., Carvalho G.A. (2023). Survival and demography of the tomato borer (*Tuta absoluta*) exposed to citrus essential oils and major compounds. Agriculture.

[15] Antunes M.D.C., Cavaco A.M. (2010). The use of essential oils for postharvest decay control. A review. Flavour Fragr. J..

[16] Zaker M. (2016). Natural Plant Products as Eco-Friendly Fungicides for Plant Diseases Control—A Review. Agriculturists.

[17] Matos L.F., da Cruz Lima E., de Andrade Dutra K., Navarro D.M.D.A.F., Alves J.L.R., Silva G.N. (2020). Chemical composition and insecticidal effect of essential oils from *Illicium verum* and *Eugenia caryophyllus* on *Callosobruchus maculatus* in cowpea. Ind. Crop. Prod..

[18] Abdelali S.K., Souttou K., Kacimi-Elhassani M., Aissaoui L., Bendachou H. (2023). Chemical composition of *Artemesia herba-alba* essential oil and its larvicidal and pupicidal effects against *Culex pipiens* (Diptera; Culicidae). Actual. Biol..

[19] Djerrad Z., Kadik L., Djouahri A. (2015). Chemical variability and antioxidant activities among *Pinus halepensis* Mill. essential oils provenances, depending on geographic variation and environmental conditions. Ind. Crop. Prod..

[20] Bouyahya A., Belmehdi O., Abrini J., Dakka N., Bakri Y. (2019). Chemical composition of *Mentha suaveolens* and *Pinus halepensis* essential oils and their antibacterial and antioxidant activities. Asian Pac. J. Trop. Med..

[21] El Omari N., Guaouguaou F.E., El Menyiy N., Benali T., Aanniz T., Chamkhi I., Bouyahya A. (2021). Phytochemical and biological activities of *Pinus halepensis* mill., and their ethnomedicinal use. J. Ethnopharmacol..

[22] Mohdeb S., Labdelli F., Bouriah N., Benouadah S., Ouarab S. (2022). Chemical study and insecticidal activity of *Pinus halepensis* Mill essential oil against *Bactrocera oleae* Adults. Fresenius Environ. Bull..

[23] Fakayode O.A., Abobi K.E. (2018). Optimization of oil and pectin extraction from orange (*Citrus sinensis*) peels: A response surface approach. J. Anal. Sci. Technol..

[24] McDonald L.L., Guy R.H., Speirs R.D. (1970). Preliminary Evaluation of New Candidate Materials as Toxicants, Repellents and Attractants against Stored Product Insects.

[25] Mazzonetto F. (2003). Efeito de pós de origem vegetal sobre *Acanthoscelides obtectus* (Say) (Coleoptera: Bruchidae) em feijão armazenado. Neotrop. Entomol..

[26] Paulraj M.G., Sahayaraj K. (2002). Efficacy of *Eclipta alba* (L.) Hassk and *Ocimum sanctum* (L.) leaves extracts and powders against *Tribolium castaneum* (Herbst) (Coleoptera: Tenebrionidae) in groundnut. Vistas of Entomological Research for the New Millenium.

[27] Ebadollahi A., Naseri B., Abedi Z., Setzer W.N., Changbunjong T. (2022). Promising insecticidal efficiency of essential oils isolated from four cultivated *Eucalyptus* species in Iran against the lesser grain borer, *Rhyzopertha dominica* (F.). Insects.

[28] Abbott W.S. (1925). A method of computing the effectiveness of an insecticide. J. Econ. Entomol..

[29] Mossa A.T.H. (2016). Green pesticides: Essential oils as biopesticides in insect-pest management. IJEST.

[30] Ukoroije R.B., Otayor R.A. (2020). Review on the bio-insecticidal properties of some plant secondary metabolites: Types, formulations, modes of action, advantages and limitations. Asian J. Res. Zool..

[31] Arab R., Lemeailbi N., Benhissen S. (2022). Repellent Activity of Essential Oils from *Artemisia herba alba* Asso. and *Teucrium polium* L. Against Tow Stored Product Insects. NVEO.

[32] Plata-Rueda A., Fiaz M., Brügger B.P., Cañas V., Coelho R.P., Zanuncio J.C., Martinez L.C., Serrão J.E. (2022). Lemongrass essential oil and its components cause effects on survival, locomotion, ingestion, and histological changes of the midgut in *Anticarsia gemmatalis* caterpillars. Toxin Rev..

[33] Saıfı R., Saıfı H., Akca İ., Benabadelkader M., Askın A.K., Belghoul M. (2023). Insecticidal and repellent effects of *Mentha longifolia* L. essential oil against *Aphis craccivora* Koch (Hemiptera: Aphididae). Chem. Biol. Technol. Agric..

[34] Bouzeraa H., Bessila-Bouzeraa M., Labed N. (2019). Repellent and fumigant toxic potential of three essential oils against *Ephestia kuehniella*. Biosyst. Divers..

[35] Torto B. (2009). Chemical signals asattractants, repellents and aggregation stimulants. Chem. Ecol..

[36] Green P.W. (2011). Insect-derived compounds affect the behaviour of *Liposcelis bostrychophila*: Effects of combination and structure. J. Stored Prod. Res..

[37] Sritabutra D., Soonwera M. (2013). Repellent activity of herbal essential oils against *Aedes aegypti* (Linn.) and *Culex quinquefasciatus* (Say.). Asian Pac. J. Trop. Dis..

[38] Sathantriphop S., Achee N.L., Sanguanpong U., Chareonviriyaphap T. (2015). effects of plant essential oils on escape response and mortality rate of *Aedes aegypti* and *Anopheles minimus*. J. Vector Ecol..

[39] Martynov V.O., Titov O.G., Kolombar T.M., Brygadyrenko V.V. (2019). Influence of essential oils of plants on the migration activity of *Tribolium confusum* (Coleoptera, Tenebrionidae). Biosyst. Divers..

[40] Adebisi O., Dolma S.K., Verma P.K., Singh B., Reddy S.G. (2019). Volatile, nonvolatile composition and biological activities of *Ageratum houstonianum* Mill. against diamondback moth, *Plutella xylostella* (L.) and aphid, Aphis craccivora Koch. Indian J. Exp. Biol..

[41] Farag M., Ahmed M.H., Yousef H., Abdel-Rahman A.H. (2011). Repellent and insecticidal activities of *Melia azedarach* L. against cotton leafworm, *Spodoptera littoralis* (Boisd.). ZNC.

[42] Nerio L.S., Olivero-Verbel J., Stashenko E. (2010). Repellent activity of essential oils: A review. Bioresour. Technol..

[43] Gad H.A., Ramadan G.R., El-Bakry A.M., El-Sabrout A.M., Abdelgaleil S.A. (2022). Monoterpenes: Promising natural products for public health insect control-A review. Int. J. Trop. Insect Sci..

[44] Zhang W.J., Yang K., You C.X., Wang C.F., Geng Z.F., Su Y., Wang Y., Du S.S., Deng Z.W. (2015). Contact toxicity and repellency of the essential oil from *Mentha haplocalyx* Briq. against *Lasioderma serricorne*. Chem. Biodivers..

[45] Hieu T.T., Jung J., Kim S.I., Ahn Y.J., Kwon H.W. (2014). Behavioural and electroantennogram responses of the stable fly (*Stomoxys calcitrans* L.) to plant essential oils and their mixtures with attractants. Pest Manag. Sci..

[46] Mendoza-García E.E., Ortega-Arenas L.D., Serrato-Cruz M.Á., Villanueva-Jiménez J.A., López-Arroyo J.I., Pérez-Pacheco R. (2019). Chemical composition, toxicity, and repellence of plant essential oils against *Diaphorina citri* (Hemiptera: Liviidae). Chil. J. Agric. Res..

[47] Devi M.A., Sahoo D., Singh T.B., Rajashekar Y. (2020). Toxicity, repellency and chemical composition of essential oils from Cymbopogon species against red flour beetle *Tribolium castaneum* Herbst (Coleoptera: Tenebrionidae). JCF.

[48] Seada M.A., Hamza A.M. (2023). Comparative morphology of sensilla of antennae, maxillary and labial palpi of adult *Rhyzopertha dominica* (F.) (Coleoptera: Bostrichidae), with specific reference to the typology and possible functions. JOBAZ.

[49] Sousa D.L., Xavier E.O., da Cruz R.C.D., de Souza I.A., de Oliveira R.A., da Silva D.C., Gualberto S.A., de Freitas J.S. (2023). Chemical composition and repellent potential of essential oil from Croton tetradenius (Euphorbiaceae) leaves against *Aedes aegypti* (Diptera: Culicidae). Biocatal. Agric. Biotechnol..

[50] Venthur H., Zhou J.J. (2018). Odorant receptors and odorant-binding proteins as insect pest control targets: A comparative analysis. Front. Physiol..

[51] Del Mármol J., Yedlin M.A., Ruta V. (2021). The structural basis of odorant recognition in insect olfactory receptors. Nature.

[52] Reinecke A., Hilker M. (2014). Plant semiochemicals–perception and behavioural responses by insects. Annu. Plant Rev. Insect-Plant Interact..

[53] Conchou L., Lucas P., Meslin C., Proffit M., Staudt M., Renou M. (2019). Insect odorscapes: From plant volatiles to natural olfactory scenes. Front. Physiol..

[54] Merritt D.M.A. (2022). Discriminating Memory: Learning and Chemosensation in *C. elegans*. Ph.D. Thesis.

[55] Dambolena J.S., Zunino M.P., Herrera J.M., Pizzolitto R.P., Areco V.A., Zygadlo J.A. (2016). Terpenes: Natural products for controlling insects of importance to human health—A structure-activity relationship study. Psyche A J. Entomol..

[56] Eesiah S., Yu J., Dingha B., Amoah B., Mikiashvili N. (2022). Preliminary assessment of repellency and toxicity of essential oils against *Sitophilus zeamais* motschulsky (Coleoptera: Curculionidae) on stored organic corn grains. Foods.

[57] Alami A., El Ouali Lalami A., Annemer S., El-Akhal F., Ez Zoubi Y., Farah A. (2023). Chemical Composition and Larvicidal Properties of Essential Oils from Wild and Cultivated *Artemisia campestris* L., an Endemic Plant in Morocco. Sci. World..

[58] Chen Y., Luo J., Zhang N., Yu W., Jiang J., Dai G. (2021). Insecticidal activities of *Salvia hispanica* L. essential oil and combinations of their main compounds against the beet armyworm *Spodoptera exigua*. Ind. Crop. Prod..

[59] Bano P., Rather M.A., Mukhtar M., Sherwani A., Ganie S. (2022). Fumigant Toxicity of *Artemisia absinthium* Essential Oil to Common Stored Product Pests. Indian J. Entomol..

[60] Ejjabraoui M., Mohamed Abdoul-Latif F., Eddabbeh F.E., Ainane A., Shybat Z.L., Ainane T. (2021). Chemical study and insecticidal activity of two species of Moroccan Pinus: *Pinus halepensis* Mill. and *Pinus pinaster* Sol. Pharmacol. Online.

[61] Li Q., Song Y. (1998). Studies on effect of several plant materials against stored grain insects. Proceedings of the Seventh International Conference on Stored-Product Protection.

[62] Wu Y., Zhang W.J., Huang D.Y., Wang Y., Wei J.Y., Li Z.H., Sun J.S., Bai J.F., Tian Z.F., Wang P.J. (2015). Chemical compositions and insecticidal activities of *Alpinia kwangsiensis* essential oil against *Lasioderma serricorne*. Molecules.

[63] Zhang J.W., Li B.Y., Lu X.X., Zheng Y., Wang D., Zhang Z., Zeng D., Du S.S. (2022). Chemical Diversity and Anti-Insect Activity Evaluation of Essential Oils Extracted from Five *Artemisia* Species. Plants.

[64] Chaudhari A.K., Singh V.K., Kedia A., Das S., Dubey N.K. (2021). Essential oils and their bioactive compounds as eco-friendly novel green pesticides for the management of storage insect pests: Prospects and retrospects. ESPR.

[65] Colovic M.B., Krstic D.Z., Lazarevic-Pasti T.D., Bondzic A.M., Vasic V.M. (2013). Acetylcholinesterase inhibitors: Pharmacology and toxicology. Curr. Neuropharmacol..

[66] Chaubey M.K. (2016). Insecticidal activities of *Cinnamomum tamala* (Lauraceae) essential oil against *Sitophilus oryzae* L. (Coleoptera: Curculionidae). Int. J. Entomol. Res..

[67] Buss E.A., Park-Brown S.G. (2002). Natural Products for Insect Pest Management.

[68] Abdelgaleil S.A.M., Gad H.A., Ramadan G.R., El-Bakry A.M., El-Sabrout A.M. (2024). Monoterpenes: Chemistry, insecticidal activity against stored product insects and modes of action—A review. Int. J. Pest Manag..

[69] Yousef H.E.B.A., El-Lakwah S.F., El Sayed Y.A. (2013). Insecticidal activity of linoleic acid against *Spodoptera littoralis* (Boisd.). EJAR.

[70] Ren Y., Shi J., Mu Y., Tao K., Jin H., Hou T. (2019). AW1 neuronal cell cytotoxicity: The mode of action of insecticidal fatty acids. J. Agric. Food Chem..

[71] Ling W., Kaliaperumal K., Huang M., Liang Y., Ouyang Z., Zhou Z., Jiang Y., Zhang J. (2022). Pomelo seed oil: Natural insecticide against cowpea aphid. Front. Plant Sci..

[72] Toews M.D., Subramanyam B. (2003). Contribution of contact toxicity and wheat condition to mortality of stored-product insects exposed to spinosad. Pest Manag. Sci..

[73] Balabanidou V., Grigoraki L., Vontas J. (2018). Insect cuticle: A critical determinant of insecticide resistance. Curr. Opin. Insect Sci..

[74] Cao J.Q., Guo S.S., Wang Y., Pang X., Geng Z.F., Du S.S. (2018). Toxicity and repellency of essential oil from *Evodia lenticellata* Huang fruits and its major monoterpenes against three stored-product insects. Ecotoxicol. Environ. Saf..

[75] Elbrense H., Gheda S. (2021). Evaluation of the insecticidal and antifeedant activities of some seaweed extracts against the Egyptian cotton leaf worm, *Spodoptera littoralis*, and the lesser grain borer *Rhyzopertha dominica*. Egypt. J. Exp. Biol..

[76] Şengül Demirak M.Ş., Canpolat E. (2022). Plant-based bioinsecticides for mosquito control: Impact on insecticide resistance and disease transmission. Insects.

[77] Lucia A., Guzmán E. (2021). Emulsions containing essential oils, their components or volatile semiochemicals as promising tools for insect pest and pathogen management. Adv. Colloid Interface Sci..

[78] He Y., Du G., Xie S., Long X., Sun G., Zhu S., Chen B. (2022). The Insecticidal Efficacy and Physiological Action Mechanism of a Novel Agent GC16 against *Tetranychus pueraricola* (Acari: Tetranychidae). Insects.

